# Detection of Biochemically Recurrent Prostate Cancer with [^18^F]DCFPyL PET/CT: An Updated Systematic Review and Meta-Analysis with a Focus on Correlations with Serum Prostate-Specific Antigen Parameters

**DOI:** 10.3390/tomography9040120

**Published:** 2023-08-15

**Authors:** Mohammad S. Sadaghiani, Sara Sheikhbahaei, Abdullah Al-Zaghal, Lilja B. Solnes, Martin G. Pomper, Jorge D. Oldan, Gary A. Ulaner, Michael A. Gorin, Steven P. Rowe

**Affiliations:** 1 The Russell H. Morgan Department of Radiology and Radiological Science, Johns Hopkins University School of Medicine, Baltimore, MD 21287, USA; 2 Department of Radiology, University of North Carolina, Chapel Hill, NC 27599, USA; 3 Molecular Imaging and Therapy, Hoag Family Cancer Institute, Irvine, CA 92633, USA; 4 Departments of Radiology, University of Southern California, Los Angeles, CA 90089, USA; 5 Department of Translational Genomics, University of Southern California, Los Angeles, CA 90089, USA; 6 Milton and Carroll Petrie Department of Urology, Icahn School of Medicine at Mount Sinai, New York, NY 10029, USA

**Keywords:** PSMA, prostate-specific membrane antigen, molecular imaging

## Abstract

[^18^F]DCFPyL is increasingly used for prostate-specific membrane antigen (PSMA) mediated imaging of men with biochemically recurrent prostate cancer (BRPCa). In this meta-analysis, which is updated with the addition of multiple new studies, including the definitive phase III CONDOR trial, we discuss the detection efficiency of [^18^F]DCFPyL in BRPCa patients. PubMed was searched on 29 September 2022. Studies evaluating the diagnostic performance of [^18^F]DCFPyL among patients with BRPCa were included. The overall pooled detection rate with a 95% confidence interval (95% CI) was calculated among all included studies and stratified among patients with PSA ≥ 2 vs. <2 ng/mL and with PSA ≥ 0.5 vs. <0.5 ng/mL. The association of detection efficiency with pooled PSA doubling time from two studies was calculated. Seventeen manuscripts, including 2252 patients, met the inclusion criteria and were used for data extraction. A previous meta-analysis reported that the pooled detection rate was 0.81 (95% CI: 0.77–0.85), while our study showed a pooled overall detection rate of 0.73 (95% CI: 0.66–0.79). An increased proportion of positive scans were found in patients with PSA ≥ 2 vs. <2 ng/mL and PSA ≥ 0.5 vs. <0.5 ng/mL. No significant difference was found in detection efficiency between those with PSA doubling time ≥ 12 vs. <12 months. Detection efficiency is statistically related to serum PSA levels but not to PSA doubling time based on available data. The detection efficiency of [^18^F]DCFPyL in men with BRPCa has trended down since a previous meta-analysis, which may reflect increasingly stringent inclusion criteria for studies over time.

## 1. Introduction

Prostate cancer (PCa) remains the most common non-cutaneous cancer among men worldwide [1]. Although the majority of patients with localized disease have a favorable response to initial treatment with either radical prostatectomy or radiation therapy, biochemical recurrence (BCR) remains relatively common and, if left untreated, can progress to incurable metastatic disease. According to the American Urological Association (AUA), BCR after radical prostatectomy is defined as two consecutive serum PSA values of ≥0.2 ng/mL after being undetectable [2], whereas BCR after radiation therapy is defined as a serum prostate-specific antigen (PSA) rise of ≥2.0 ng/mL above the nadir [3]. A key step in selecting the most appropriate form of treatment for BCR is to determine the anatomical distribution and volume of a patient’s disease. Compared to standard clinical parameters alone, the use of molecular imaging with [^18^F]FACBC PET/CT to guide salvage radiation therapy has been shown to improve outcomes for men with BCR after radical prostatectomy [4]. In the time since completing that trial, molecular imaging with PET radiotracers targeting prostate-specific membrane antigen (PSMA) has emerged as the standard of care for men with BCR. Although a trial is currently underway evaluating the benefits of using PSMA-targeted PET imaging to guide salvage treatment in men with BCR [5,6], most clinicians have already begun using this form of molecular imaging to inform decisions regarding the care of patients with BCR.

Prostate-specific membrane antigen (PSMA) is a transmembrane protein that is localized within the cytoplasm in benign prostatic cells, with malignant transformation resulting in expression on the surface of cells with a resultant large extracellular domain [7]. It is known that PSMA Expression is 100- to 1000-fold higher in PCa versus benign cells, and that small molecules capable of binding to the catalytic domain of the PSMA protein are rapidly internalized within PCa cells [8]. In recent years, it has become possible to not only detect but also treat PCa using radiolabeled small molecules targeting PSMA. For the purpose of diagnostic imaging, the two most commonly used PET imaging agents for targeting PSMA are [^18^F]DCFPyL and [^68^Ga]PSMA-11.

In contrast to gallium-68, fluorine-18 has a longer half-life and better spatial resolution, which makes it an ideal agent for cancer detection [9]. Because of these properties, [^18^F]DCFPyL has emerged as the radiotracer of choice among most clinicians, at least in the United States. A prior meta-analysis of the available medical literature up to December 2020 showed that the agent is associated with a relatively high detection rate among patients with BCR and that the rate of prostate cancer detection is highly dependent on the patient’s serum prostate-specific antigen (PSA) level [10]. In this report, we provided an updated systematic review and meta-analysis of the diagnostic performance of this agent among patients with BCR following definitive local treatment for PCa, with a particular emphasis on correlations to PSA parameters. This updated meta-analysis incorporates the results of several key clinical trials [11,12,13] that have been published since the time of the earlier report and includes data on three times as many patients.

## 2. Materials and Methods

This study adhered to the Preferred Reporting Items for Systematic Reviews and Meta-Analyses (PRISMA) 2020 checklist. A comprehensive search of the literature was performed on 29 September 2022 using the MEDLINE database. Clinical studies in humans evaluating the diagnostic performance of [^18^F]DCFPyL among patients with BRPCa that were written in the English language were included. The overall pooled detection rate with 95% confidence intervals (95% CIs) was calculated among all included studies. In addition, we compared the pooled detection rates among patients with PSA ≥ 2 ng/mL versus those with PSA < 2 ng/mL, as well as the pooled detection rates in patients with PSA < 0.5 ng/mL versus those with PSA ≥ 0.5 ng/mL. We also stratified the detection rate according to the location of malignant tissue, including local recurrence (prostate bed and seminal vesicles), locoregional lymph node involvement, osseous metastases, and visceral metastases. Meta-analysis for calculation of the pooled proportion of patients with positive findings was performed with R version 4.2.2 (31 October 2022) using a meta package (version 6.0-0) [14].

We also compared the pooled proportion of positive scans in two studies [15,16] according to PSA doubling time < 12 months versus those ≥ 12 months using Review Manager (RevMan) version 5.4. We performed the meta-analysis based on a random-effects model. *I^2^* was calculated to quantify the heterogeneity.

To evaluate the contribution of possible covariates in the heterogeneity, meta-regression analysis was performed with R version 4.2.2 using meta package version 6.0-0 for overall detection. Logit transformation with the inverse variance method was used to perform a meta-analysis of proportions. In addition, Funnel plots were used to assess publication bias. The overall quality of the studies was evaluated based on the revised “Quality Assessment of Diagnostic Accuracy Studies” tool (QUADAS-2) using Review Manager (RevMan) version 5.4 [17]. QUADAS-2 evaluates four domains (patient selection, index test, reference standard, and flow and timing), and each domain was assessed in terms of risk of bias. In addition, the first three domains were considered as a measure to check applicability. The reference standard was considered histopathologic correlation, and if the methods section of an included study clarified that at least some of the lesions were evaluated with pathology, the study was considered a low risk of bias for the reference standard. Otherwise, it was labeled as high risk for reference standards.

## 3. Results

**Figure 1:**
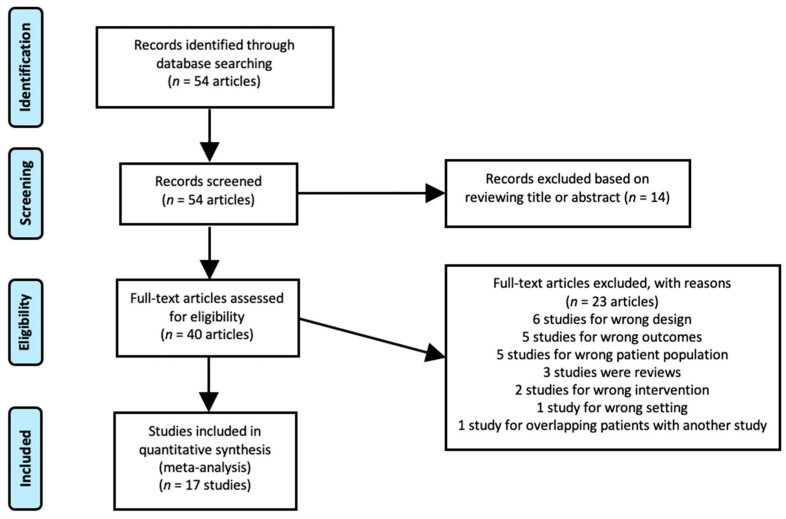
PRISMA diagram of the study.

Fifty-four articles were reviewed individually against our inclusion criteria. Fourteen studies were omitted by reviewing the title or abstract. Full texts of the remaining 40 studies were reviewed, and 22 studies were excluded. As such, a total of 17 manuscripts, including 2252 patients, met the inclusion criteria and were used for data extraction (Figure 1 and Table 1). Two studies by Mena et al. [12,18] showed significant overlap, and only the later study with a greater number of cases was included in the meta-analysis to assess overall detection [12]. However, the smaller and earlier study published in 2020 [18] was used to calculate the pooled proportion of positive cases stratified based on the location of disease, the information of which was not provided in the larger study.

**Figure 2:**
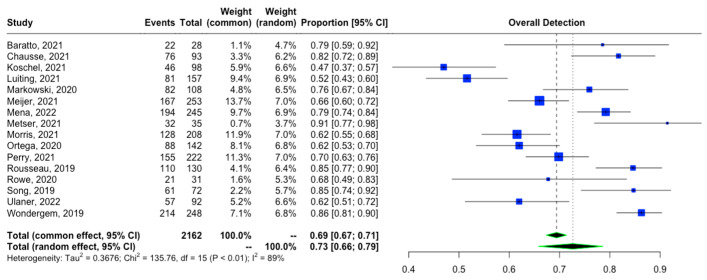
Overall detection rate of [^18^F]DCFPyL in patients with BRPCa [11,12,13,15,16,19,20,21,22,23,24,25,26,27,28,29].

**Figure 3:**
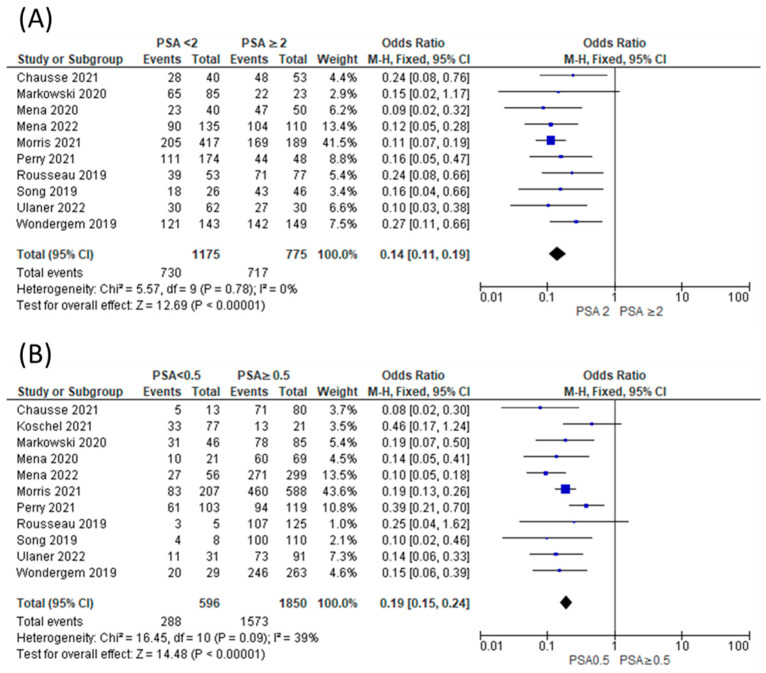
Comparison of proportion of patients showing positive [^18^F]DCFPyL scans among those with PSA < 2 compared to those with PSA ≥ 2 (**A**) [11,12,13,15,16,20,26,27,29] and among those with PSA < 0.5 versus PSA ≥ 0.5 (**B**) [11,12,13,15,16,20,21,26,27,29].

**Figure 4:**
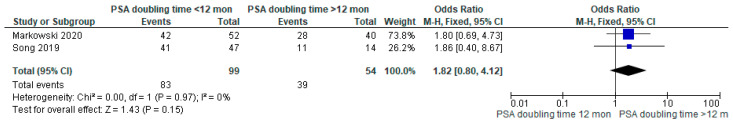
Comparison of proportion of patients showing positive [^18^F]DCFPyL scan among those with PSA doubling time <12 months versus >12 months [15,16].

The proportion of patients showing positive scans, along with the total PSA values, PSA doubling time, and location of PSMA avid lesions, were extracted. A forest plot representing the pooled data from all included studies showing the proportion of patients with positive scans is shown in Figure 2. The overall detection rate between studies was markedly heterogeneous (range: 0.47–0.91, *I*^2^: 89%) and was influenced considerably by the absolute PSA level of imaged patients (Figure 3). Higher PSA values were associated with a significantly higher detection rate. In addition, stratifying the data based on PSA values resulted in an improvement of the heterogeneity, for example, a comparison of the proportion of patients showing the proportion of positive [^18^F]DCFPyL scans among those with PSA < 2 compared to those with PSA ≥ 2 (Figure 3A) showed *I^2^* of 0 and among those with PSA < 0.5 versus PSA ≥ 0.5 showed *I*^2^ of 44%. In contrast, we did not observe a difference in the cancer detection efficiency with stratification by PSA doubling time of ≤12 versus >12 months (Figure 4).

**Figure 5:**
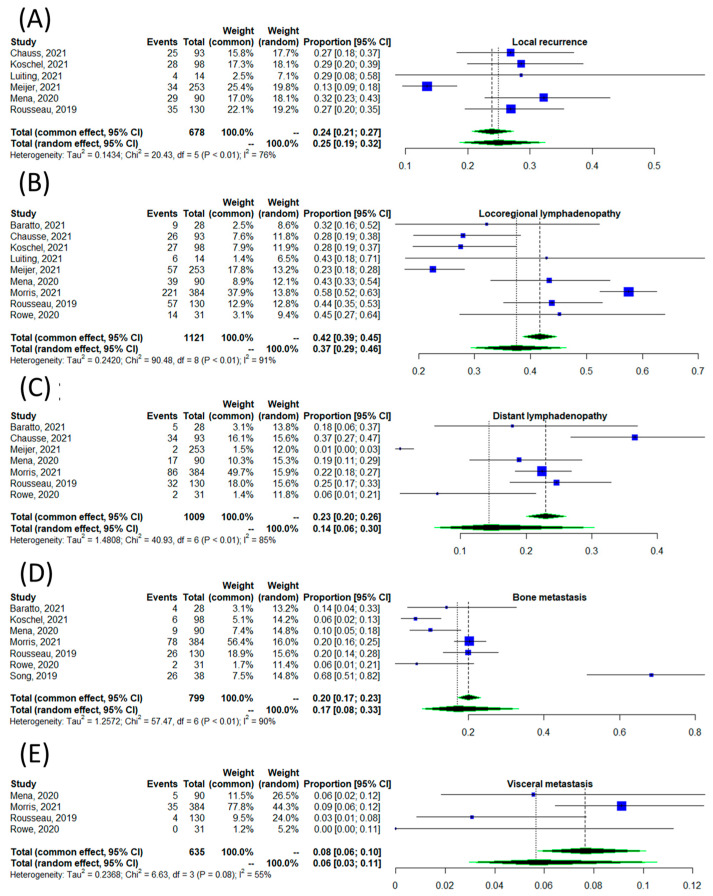
The detection rate of [^18^F]DCFPyL scan according to the site of disease, including local recurrence (**A**) [18,20,21,22,23,27], locoregional lymphadenopathy (**B**) [11,18,19,20,21,22,23,27,28], distant lymphadenopathy (**C**) [11,18,19,20,23,27,28], osseous metastases (**D**) [11,16,18,19,21,27,28], and visceral metastases (**E**) [18,20,21,22,23,27].

Figure 5 illustrates the pooled proportion of positive scans stratified according to different anatomical regions, including local recurrence, which itself is comprised of prostate bed and seminal vesicles (Figure 5A), locoregional lymph node involvement (Figure 5B), distant lymph node involvement (Figure 5C), bone metastases (Figure 5D), and visceral metastases (Figure 5E). The pooled proportions and 95% CIs of positive scans by anatomical site were 0.26 (95% CI: 0.19–0.32), 0.37 (95% CI: 0.29–0.46), 0.14 (95% CI: 0.06–0.30), 0.17 (95% CI: 0.08–0.33), and 0.08 (95% CI: 0.06–0.10), respectively. These results were associated with moderate to high heterogeneity; the lowest heterogeneity in detection rate was among those with visceral metastasis (*I*^2^ = 55%), and the highest heterogeneity was among those with locoregional lymphadenopathy (*I*^2^ = 99%).

To further assess the heterogeneity, a meta-regression was performed using histopathologic validation as the standard of truth. We found that ten studies did not provide histopathologic validation, while seven studies verified at least a portion of lesions with radiotracer uptake via tissue sampling (Table 1). Only one study compared all imaging findings with the ground truth of histopathology [13]. Meta-regression did not show a statistically significant difference.

**Figure 6:**
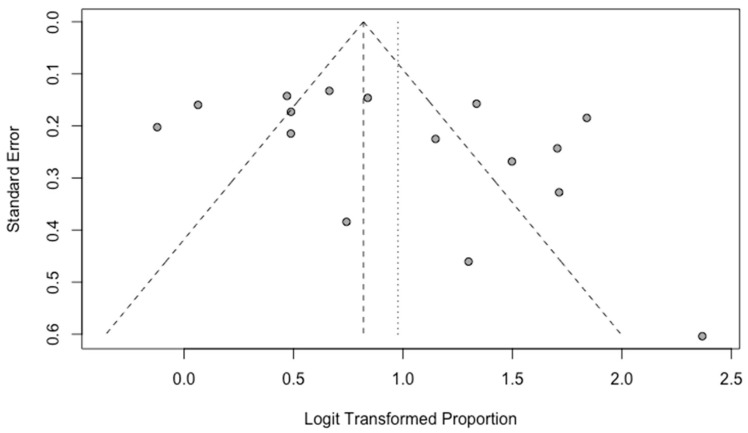
Funnel plot for the studies included in the evaluation of the overall detection rate.

A funnel plot was used for the evaluation of possible publication bias. By visual inspection, there is no subjective asymmetry among the studies in overall detection rate evaluation (Figure 6).

**Figure 7:**
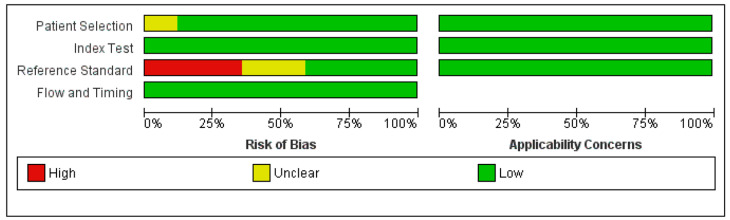
Risk of bias and applicability concerns summary based on QUADAS-2.

Assessment of the risk of bias using QUADAS-2 showed that, in addition to the reference standard, overall, the studies showed a low risk of bias (Figure 7). Six studies did not include any pathologic correlation as a measure of the reference standard, and four studies did not clarify their approach.

## 4. Discussion

In this systematic review and meta-analysis, we provide updated estimates of the pooled sensitivity of [^18^F]DCFPyL PET/CT for detecting sites of disease in men with BRPCa following local definitive therapy. This is also the first meta-analysis to include the definitive phase III trial (CONDOR) that helped establish [^18^F]DCFPyL as a standard of care in men with BRPCa. Additionally, we stratified the data according to the PSA level and PSA doubling time and showed that the proportion of positive scans is significantly higher among those with PSA values ≥ 2 ng/mL versus <2 ng/mL and among those with PSA values ≥ 0.5 versus <0.5. These findings confirm that higher PSA levels improve detection efficiency. This may impact decision-making regarding the timing of when to image a patient, but also demonstrates that there is no cut-off below which there is not at least a moderate detection efficiency. In contrast, the rates of cancer detection by PSA doubling time did not show a significant difference at the conventional *p*-value threshold of 0.05. Although this finding may represent reality, it is more likely that our study was simply underpowered to detect an association between this parameter and cancer detection.

This meta-analysis is an update to a previously published study by Sun et al. [10], which included nine studies in their meta-analysis of [^18^F]DCFPyL overall detection rate in BCR. Our study compiled 17 studies for this purpose, including the most up-to-date studies during the last year. Sun et al. reported that the pooled detection rate was 0.81 (95% CI: 0.77–0.85), while our study showed a pooled overall detection rate of 0.73 (95% CI: 0.67–0.79), which may reflect an overall trend toward a more stringent definition of BCR in study inclusion criteria. In addition, we noticed an increase in heterogeneity, with *I*^2^ being 53.2% in the prior study and 90.0% in ours. Subjective evaluation of the outliers on the forest plot (Figure 2) showed that the possible contributors are the studies by Koschel et al. [18] and Luiting et al. [19]. In addition, heterogeneity was further assessed with meta-regression based on the presence of histopathologic validation as the standard of truth. The bubble plot in Figure 6 demonstrated that the difference in the standard of truth at least partially could explain the heterogeneity.

Stratification of the results according to the site of disease was comparable to the findings of the study by Sun et al. [10]. Similar to our study, Sun et al. also found regional lymphadenopathy to be the most common site of disease and osseous metastasis as the least common site of metastasis. The slightly higher proportions in their study could be at least partly related to regression to the mean.

Limitations of the current analysis include the high heterogeneity among trials and the fundamental differences in truth standards that were utilized. The majority of the studies did not stratify the results based on prior radiation and/or radical prostatectomy, which likely contributed to the high heterogeneity observed in our results. Nonetheless, this study serves as an important update on the importance of PSMA-targeted imaging with [^18^F]DCFPyL in men with BRPCa.

## 5. Conclusions

Our meta-analysis shows that the detection efficiency of [^18^F]DCFPyL in men with BRPCa remains overall high but has trended down from earlier estimates in the literature, leading to increased heterogeneity in the reporting of this outcome. This observation may be related to the use of increasingly stringent inclusion criteria in studies over time, such as the requirement for negative conventional imaging as in the CONDOR trial. Another factor may be the trend toward imaging of men with lower PSA values in more recent studies. Indeed, our analysis has shown that PSA level at the time of imaging is unmistakably linked to cancer detection rates, whereas PSA doubling time was not. Finally, differences in the required truth standard for test positivity have likely also contributed to the high degree of heterogeneity in the literature, and this must be taken into account when comparing cancer detection rates across studies.

## Figures and Tables

**Table 1:** Characteristics of the included studies.

Author, Year	Radiotracer Dose	Time Interval between Injection and Scanning	PSA	Standard of Truth	# Patients
Baratto, 2021 [19]	270.1 to 366.3 MBq (333 ± 25.9 MBq)	60 to 120 min (81.2 ± 17 min)	Mean 5 ng/mL	At least part of the data was validated by histopathology	28
Chaussé, 2021 [20]	9 ± 1 mCi (333 ± 37 MBq)	60–90 min	Median 2.27 ng/mL	At least part of the data was validated by histopathology	93
Koschel, 2021 [21]	250 MBq ± 50 MBq	120 min	Median 0.32 ng/mL	No histopathologic validation	98
Luiting, 2021 [22]	Not provided	Not provided	Median 0.30 ng/mL (IQR 0.23–0.70)	No histopathologic validation	157
Markowski, 2020 [15]	333 MBq (9 mCi)	60 min	Median 0.7 ng/mL (IQR 0.3–1.8)	No histopathologic validation	108
Meijer, 2021 [23]	311 MBq (interquartile range (IQR) 301–322 MBq)	120 min	The median PSA level at the time of the PET scan post-RARP was 0.5 ng/mL (IQR 0.2–1.1), median 0.9 ng/mL (IQR 0.3–2.8) in patients post-RARP + SRT, and median 2.8 ng/mL (IQR 1.3–5.6) in patients post-EBRT	No histopathologic validation	253
Mena, 2020 [18]	Mean 299.9 ± 15.5 MBq, 8.09 mCi (6.2–8.8 mCi)	-	Median 2.5 ng/mL (range 0.21–35.5)	At least part of the data was validated by histopathology	90
Mena, 2022 [12]	Mean 296 ± 33.3 MBq [8.0 ± 0.9 mCi]; range, 207.2–325.6 MBq (5.6–8.8 mCi)	1–2 h	Median 1.6 ng/mL (range, 0.2–35.5)	No histopathologic validation	245
Metser, 2021 [24]	327 (±16.7)	110.2 (±13.9) min	Median 3.0 mg/mL (range 0.5–43.3)	At least part of the data was validated by histopathology	35
Morris, 2021 [11]	MBq median 349 range (277–410), mCi 9.42 (7.49–11.07)	median 79 range (59–115)	Median 0.8 ng/mL	At least part of the data was validated by histopathology	208
Ortega, 2020 [25]	335.5 MBq (range, 223–376 MBq)	approximately 120 min (mean, 115.1 min; range, 83–168 min)	Median 3.69 mg/L (range, 0.55–49.9 mg/L)	No histopathologic validation	142
Perry, 2021 [26]	250 MBq (±50 MBq	120 min (±10 min)	Median 0.51 ng/mL (range, 0.08–58.9)	No histopathologic validation	222
Rousseau, 2019 [27]	The administered activity was scaled by body weight (range, 237–474 MBq), allowing a 10% variation in target activity.	120 min	Mean ± SD 5.20 ng/mL ± 6.50	No histopathologic validation	130
Rowe, 2020 [28]	333 MBq (9 mCi)	60 min	Median 0.4 ng/mL (range, 0.2–28.3)	No histopathologic validation	31
Song, 2019 [16]	Mean 338.8 ± 25.3	60 min	Median 3.0 ng/mL (range, 0.23 to 698.4 ng/mL)	At least part of the data was validated by histopathology	72
Ulaner, 2022 [13]	333 MBq (9 mCi) ± 10%	60 min	Median 0.7 ng/mL (range, 0.2–38.9	All data was validated by histopathology	92
Wondergem, 2019 [29]	311 MBq	120 min	_	No histopathologic validation	248

Abbreviations, IQR: interquartile range, RARP: robot-assisted radical prostatectomy, SRT: salvage radiotherapy.

## Data Availability

No new data were generated in this study.
